# What is known and unknown about the role of neuroendocrine genes *Ptprn* and *Ptprn2*

**DOI:** 10.3389/fendo.2025.1531723

**Published:** 2025-01-24

**Authors:** Stanko S. Stojilkovic, Srdjan J. Sokanovic, Stephanie Constantin

**Affiliations:** Section on Cellular Signaling, The Eunice Kennedy Shriver National Institute of Child Health and Human Development, Bethesda, MD, United States

**Keywords:** PTPRN, PTPRN2, GnRH neurons, kisspeptinergic neurons, pancreatic β-cells, gonadotrophs, melanotrophs

## Abstract

The protein tyrosine phosphatase receptors N and N2 are encoded by the *Ptprn* and *Ptprn2* genes expressed in neuroendocrine cells of the hypothalamus, pituitary gland, and diffuse neuroendocrine system, including the pancreas, lung, and intestine. Unlike other members of the protein tyrosine phosphatase receptor family, PTPRN and PTPRN2 lack protein tyrosine phosphatase activity due to mutation of two residues in their intracellular catalytic domains. However, during evolution these proteins acquired new cellular roles beyond tyrosine dephosphorylation in the centralized and diffuse neuroendocrine systems. Here we discuss the current understanding and lack of information about the actions of these proteins, focusing on neuroendocrine cells of the hypothalamus, pituitary, and pancreas.

## Introduction

Protein tyrosine phosphatase receptors (PTPRs), a family of proteins encoded by 21 genes in humans, are signaling molecules composed of an extracellular domain, a transmembrane domain, and an intracellular domain. The diverse extracellular domain shares homology with cell adhesion molecules and encompasses a wide range of physiological functions, the transmembrane domain makes them a type of membrane receptor protein and the intracellular domain contains either one or two highly conserved intracellular phosphatase domain (1, 2). Protein tyrosine phosphatase receptor type N (PTPRN, also known as IA-2 and ICA512) and PTPRN2 (also known as IA-2β and phogrin) are atypical members of this protein family. PTPRN consists of 979 amino acids encoded by the *Ptprn* gene, located on human chromosome 2q35, while PTPRN2 consists of 986 amino acids, and its *Ptprn2* gene is located on human chromosome 7q36 (3).

Comparison of the amino acid sequences of human, mouse, and rat PTPRN revealed that the intracellular domain is over 97% conserved in these species, while the extracellular domain is 80–90% conserved. The intracellular domain of PTPRN2 is 92% conserved in human, mouse and rat, whereas the extracellular domain shows only 50–60% identity (3). PTPRN and PTPRN2 share 74% identity within the intracellular, but only 27% in the extracellular domains (4–6). A splice variant of human *Ptprn* lacking exon 13 encoding the transmembrane region (7, 8), as well as three splice transcripts of human *Ptprn2* (9), have been detected in the pancreas. The extracellular domain of PTPRN and PTPRN2 proteins (hereinafter referred to as PTPRNs) show partial homology with the extracellular domain of regulated endocrine specific protein 18, which is another protein marker of neuroendocrine cells (10–12), indicating a potential physiological significance for their function (13).

## PTPRNs are expressed in neuronal and neuroendocrine cells

### Central and peripheral nervous system

Western blot analysis and physiological responses suggest that PTPRN is expressed in hippocampal tissues in mice (14, 15). PTPRN expression has also been detected in autonomic nerve fibers and ganglia (16, 17). However, others have argued that PTPRN immunoreactivity is below detectable level in the hippocampus, cerebral cortex, cerebellum, striatum, and thalamus (16). *In situ* hybridization, western blot, and immunohistochemical analyses revealed PTPRN2 expression in the cerebral cortex, hippocampus, thalamus, choroid plexus, Purkinje cells, granular layer of the cerebellum, and medulla oblongata (18, 19). Immunostaining and immunohistochemical evidence also support PTPRN2 expression in hippocampal interneurons and suggest the existence of molecularly distinct populations of secretory vesicles in different types of inhibitory neurons (20).

### Neuroendocrine regions of the brain

The hypothalamus is a central neuroendocrine hub that expresses PTPRN and PTPRN2 proteins and mRNAs (16, 18, 21–23). Within the hypothalamus, *Ptprn* and *Ptprn2* were detected in cells of the arcuate and periventricular nuclei (22) as well as in gonadotroph-releasing hormone (GnRH) neurons (24, 25), and *Ptprn*+*Ptprn2* deletion affected suprachiasmatic function (17). A high level of PTPRN immunoreactivity was detected in the amygdala, which is also considered a neuroendocrine region of the brain. The highest levels of PTPRN immunoreactivity were observed in the infundibular tract, which contains the axons of hypothalamic vasopressin and oxytocin secreting neurons that terminate in the posterior pituitary (16).

### Pituitary gland

*Ptprn* was originally cloned from the bovine pituitary (26). The rodent pituitary also expresses *Ptprn* and *Ptprn2* (18, 19, 22, 23, 27) as well as immortalized pituitary cells (28). Single cell RNA sequencing experiments with freshly dispersed rat pituitary cells revealed that these genes are expressed in hormone-producing corticotrophs, melanotrophs, gonadotrophs, thyrotrophs, somatotrophs, and lactotrophs, but not in folliculostellate cells and pituicytes (29, 30). Because pituicytes are the resident cells of the posterior pituitary, the finding that PTPRN is detected in the posterior pituitary (16) suggests that nerve endings of hypothalamic vasopressin and oxytocin neurons express this protein.

### Diffuse neuroendocrine system

In addition to neuroendocrine brain and pituitary gland, neuroendocrine cells can be found as single cells or small groups of cells scattered throughout the parenchymal surface epithelium of various tissues, including the pancreas, lung, and intestine. PTPRN is present in alpha, beta, and delta cell of the pancreas, chromaffin cells of the adrenal medulla, and thyroid C cells, also known as parafollicular cells, which are calcitonin-secreted neuroendocrine cells (16, 19, 21, 31). Immunohistochemical analysis also indicated PTPRN expression in rat gastrointestinal neuroendocrine cells (19, 32). *Ptprn* is also expressed in human lung cancer cell lines with a neuroendocrine phenotype (33).

## PTPRNs are pseudophosphatases that exhibit other cellular functions

Unlike other PTPR members, which have a tandem phosphatase domain, PTPRNs have a single phosphatase domain (34). Furthermore, the structure of the catalytic domain is altered in PTPRN and PTPRN2, suggesting that both proteins are pseudophosphatases. According to the bioinformatic definition, a pseudophosphatase is a member of phosphatase family that contains a mutation that predicts impairment or loss of its catalytic activity, regardless of whether this protein is enzymatically active or not (35). However, neither PTPRN nor PTPRN2 exhibit tyrosine phosphatase activity (36, 37). Furthermore, the enzymatic portion of PTPRNs can heterodimerize with another PTPRs and cause a 20% decrease in enzyme activity (38). However, the biological roles of PTPRNs have been investigated for several decades and have provided strong evidence that these proteins have acquired novel cellular roles beyond tyrosine dephosphorylation. In general, the sequence similarity between PTPRN and PTPRN2 suggests some levels of redundancy. Consequently, the effects on neuroendocrine cells are enhanced or observed only in double knockout (DKO) mice (39).

Loss of tyrosine phosphatase activity of PTPRNs due to mutation of two residues in the catalytic domain does not exclude the possibility that these proteins act as enzymes for other substrates. To date, no replacement substrate for PTPRN has been identified, but PTPRN2 has been reported to be able to dephosphorylate specific inositol phospholipids, including PI(3)P, PI(4,5)P2, but not PI(3,4,5)P3. When the transmembrane form of PTPRN2 was overexpressed in mammalian cells, it reduced plasma membrane PI(4,5)P2 levels in a dose-dependent manner (40). Other have reported that PTPRN2 and phospholipase C beta enzymatically reduce plasma membrane PI(4,5)P2 levels in metastatic breast cancer cells. They also found that the expression of these genes was increased in these cells, which coincided with human metastatic relapse. The authors further suggested that depletion of PI(4,5)P2 by these enzymes releases the PI(4,5)P2‐binding protein cofilin into the cytoplasm where it increases cellular migration and metastatic capacity (41). However, we observed no significant changes in InsP3-dependent calcium oscillations in gonadotrophs from DKO mice, which argues against a physiologically significant reduction in phospholipase C activity (23).

## Cell type-specific role of PTPRNs in hormone secretion by exocytosis

Several lines of research with pancreatic β-cells have shown that the secretory pathway is affected by deletion of *Ptprn* and/or *Ptprn2*. These genes appear to be required to accumulate normal levels of insulin-containing vesicles and prevent their degradation (42). Global knockout of *Ptprn* led to impaired glucose-mediated insulin secretion (43), whereas overexpression of *Ptprn* in an insulinoma cell line led to increased insulin secretion (42). *Ptprn2* knockout mice also show impaired glucose tolerance and reduced glucose-induced insulin secretion but was not sufficient to prevent the development of diabetes (27). However, the role of PTPRNs in exocytosis appears to be specific to β-cells. Renin release from dense core vesicles of neuroendocrine juxtaglomerular granular cells is not directly inhibited by DKO, but reflects reduced catecholamine release from sympathetic nerve endings (17). In female but not in male mice, it was originally suggested that DKO inhibits the accumulation and secretion of the pituitary gonadotropins luteinizing hormone (LH) and follicle-stimulating hormone, leading to infertility (27). Subsequent studies have shown that this is not the case for these hormones, that the exocytotic pathway of other anterior pituitary cells is also intact, and that the levels of hormones secretes by melanotrophs from the intermediate pituitary lobe are higher in DKO animals (see below).

Immunocytochemistry performed on cultured cells suggests that PTPRN is colocalized with neurosecretory granules and it is not a resident plasma membrane protein (16). PTPRN2 has also been reported to be enriched in the membranes of β-cell secretory granules (44). Proteomics analysis also revealed the presence of PTPRN and PTPRN2, as well as peptidyl-glycine-α-amidating monooxygenase (PAM), a neuropeptide processing enzyme (45), in insulin secretory granules (46). PTPRN has also been identified in the secretory granules of chromaffin cells (47). Expression of a fusion construct between PTPRN2-enhanced green fluorescent protein in β-cells and pheochromocytoma PC12 cells revealed the presence of this chimera in dense-core secretory granules (48, 49). PTPRN has also been reported to tether insulin secretory granules to actin microfilaments via its association with the adapter protein syntrophin beta 2 (50, 51). The same group also reported that the F-actin modifier villin-1 regulates insulin granule dynamic and exocytosis downstream of PTPRN (52). However, PTPRNs were not detected in pituitary corticotroph dense core vesicles (53) and TT endocrine cells (54), unlike PAM, the typical enzyme for this organelle. This may provide a rationale for the lack of effects of DKO on pituitary corticotroph secretion. Furthermore, *Sntb2* encoding syntrophin beta 2, and *Vil1* encoding Villin-1, are unlikely to play this role in pituitary hormone release because these genes are not expressed or are below detection by single cell RNA sequencing in hormone-producing cells (30).

## Common roles of PTPRNs in neuroendocrine cells

Solimena’s laboratory proposed the association of PTPRN with insulin secretory granules not only to explain the initiation of the exocytotic process, but also to have a post-exocytotic functions. First, they reported that exocytosis of secretory granules leads to insertion of PTPRN in the plasma membrane, which promotes calcium-dependent cleavage of its cytoplasmic domain by mu-calpain. It has been suggested that this cleavage results in the generation of a cytosolic fragment of PTPRN that is targeted to the nucleus, causing upregulation of insulin gene expression. Therefore, this new pathway links regulated exocytosis to the control of gene expression and suggests that calcium acts as a dual signal: it triggers exocytosis and activates the retrograde pathway (55). Second, the same group proposed signal transducer and activator of transcription 5 (STAT5) as the binding domain for the cytosolic fragment of PTPRN and described the synergy of glucose and growth hormone signaling (56). Third, the C-terminal fragment of PTPRN has been proposed to promote β-cell proliferation by linking signaling by STAT3 and STAT5 (57). Consistently with these findings, effects of DKO on gene expression and/or cell proliferation have also been observed in neuroendocrine cells of the hypothalamus and pituitary gland (see below). Furthermore, knockout of *Ptprn* has been reported to reduce, whereas overexpression of PTPRN increases proliferation and migration of glioma cells (58). PTPRN2 has also been suggested to play a role in other types of cancers (59).

## The role of PTPRNs in hypothalamic-pituitary-gonadal function

Early work with the pituitary gland suggested that DKO directly affects gonadotroph functions, particularly in females but not in males. In parallel with the β-cell secretion model, it has been suggested that PTPRNs are required for LH secretion, and DKO causes a lack of LH surge and ovulation. Therefore, PTPRNs in female gonadotrophs have been described as crucial for the structure and function of the dense core secretory vesicles, implying sexual dimorphism in exocytotic LH release (27). However, proteomics analysis of dense core secretory vesicles was not performed in pituitary cells to elucidate the presence/absence of PTPRNs in dense core secretory vesicles in female/male gonadotrophs. In addition, the authors did not examine the status of hypothalamic neurosecretory neurons that control pituitary gonadotroph functions.

Pituitary gonadotroph gene expression and hormone secretion are controlled by GnRH-secreting neurons (60), whose function is critically dependent on connections with kisspeptinergic neurons (61). GnRH is released in a pulsatile manner in females and males, causing oscillatory release of LH, a secretory pattern required for gonadal spermatogenesis/oogenesis and steroidogenesis (62). Pulsatile GnRH/LH release is driven by “GnRH pulse generator”, a neuronal assembly in the arcuate nucleus of the hypothalamus (63), which consist of kisspeptin-secreting neurons that control the distal processing of GnRH neurons and their secretion at the median eminence (64). The pulsatile release of GnRH/LH is sufficient for male fertility, but female fertility also depends on the sustained release of GnRH called the surge, which is necessary for ovulation (65). A distinct population of kisspeptin neurons is located in the rostral periventricular region of the third ventricle (RP3V) and stimulates the cell bodies of GnRH neurons to release GnRH, which causes the LH surge necessary for ovulation (65). Both GnRH and kisspeptin neurons also express *Ptprn*+*Ptprn2* (24, 25), so DKO may affect their functions.

**Figure 1:**
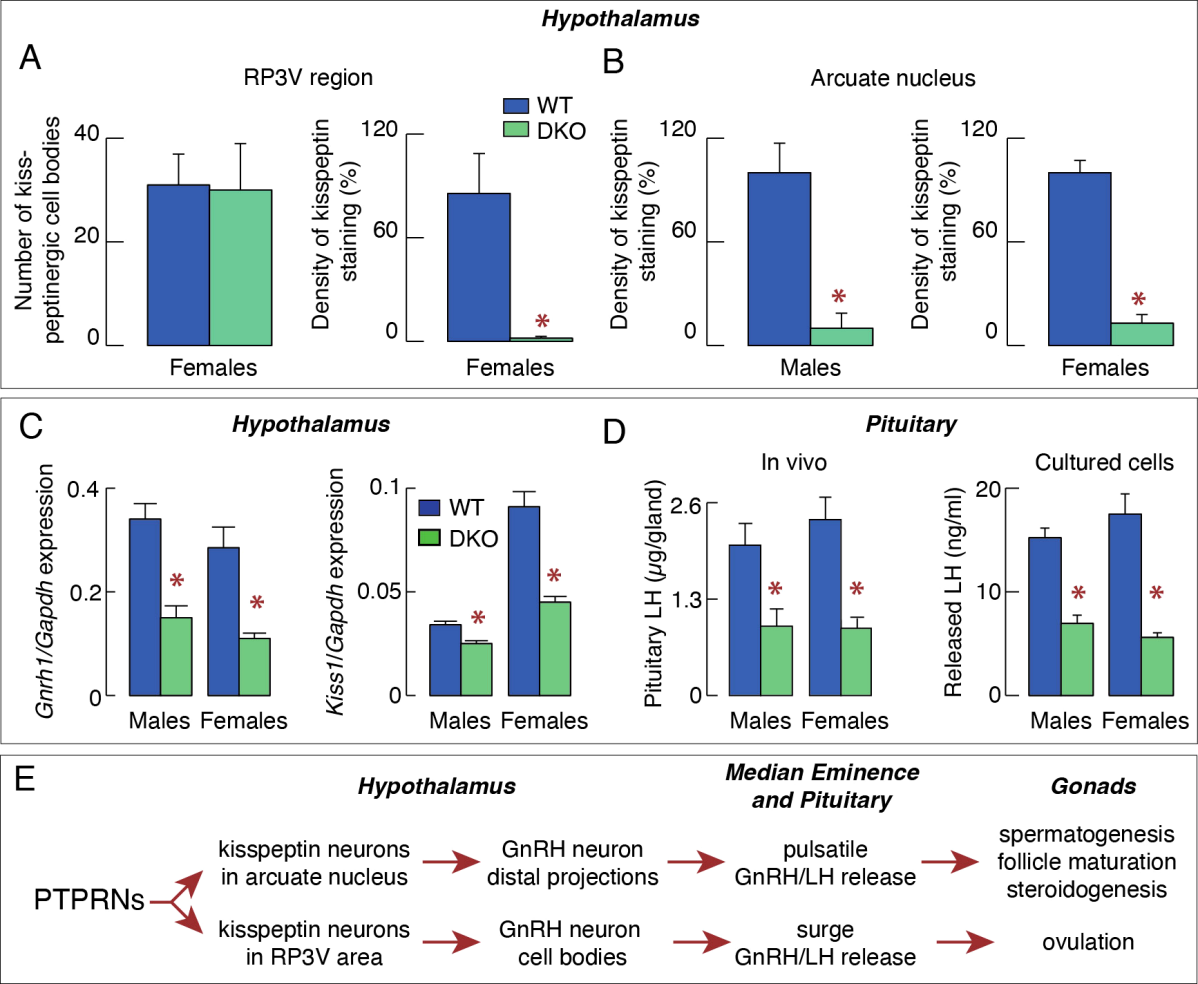
PTPRNs contribute to the control of reproduction by stimulating kisspeptin-GnRH secreting neurons. **(A)** Quantification of kisspeptinergic cell bodies (left) and fiber densities (right) in the RP3V region of WT and DKO females. **(B)** Quantification of kisspeptinergic fiber densities in the arcuate nucleus of WT and DKO males and females. **(C)** Inhibition of expression of *Gnrh1* (left) and *Kiss1* (right) in DKO animals. **(D)** Inhibition of pituitary and serum luteinizing hormone (LH) in DKO mice. **(E)** Schematic representation of the proposed stimulatory effect of PTPRN on the hypothalamic-pituitary-gonadal axis by increasing the synthesis and release of kisspeptin, which influences the pulsatile and surge release of GnRH/LH, the former being responsible for spermatogenesis, follicle maturation and steroidogenesis, and later for ovulation. Asterisks indicate significant differences between pairs, P < 0.01 Asterisks.

In a recent study (23), we showed that the density of kisspeptin staining was significantly reduced in both the arcuate nucleus and the RP3V region of DKO mice (**Figures 1A, B**). Moreover, the expression of *Gnrh1* and *Kiss1* was decreased in the hypothalamic tissue of DKO animals (**Figure 1C**). Expression of the pituitary gonadotroph-specific genes *Lhb*, *Fshb*, and *Gnrhr* was also significantly reduced in females and males (23). These changes were accompanied by significantly reduced pituitary LH accumulation and released in both females and males, arguing against sexual dimorphism at the pituitary level (**Figure 1D**). Significant changes in ovarian steroidogenesis and gene expression were also observed in DKO females, leading to the delay in puberty and female reproductive organ development (23). Others have also reported a delay in onset of puberty in *Ptprn2*-only knockout females (22). Finally, DKO females were in constant diestrus, indicating a lack of ovulation, in contrast to control females that had a 4–5-day estrous cycle. However, no changes were observed in testicular steroidogenesis and spermatogenesis, and seminal vesicles development in DKO males (23). The interpretation of these findings is summarized in the scheme shown in **Figure 1E**.

## The role of PTPRNs in hypothalamic-pituitary-adrenal function

**Figure 2:**
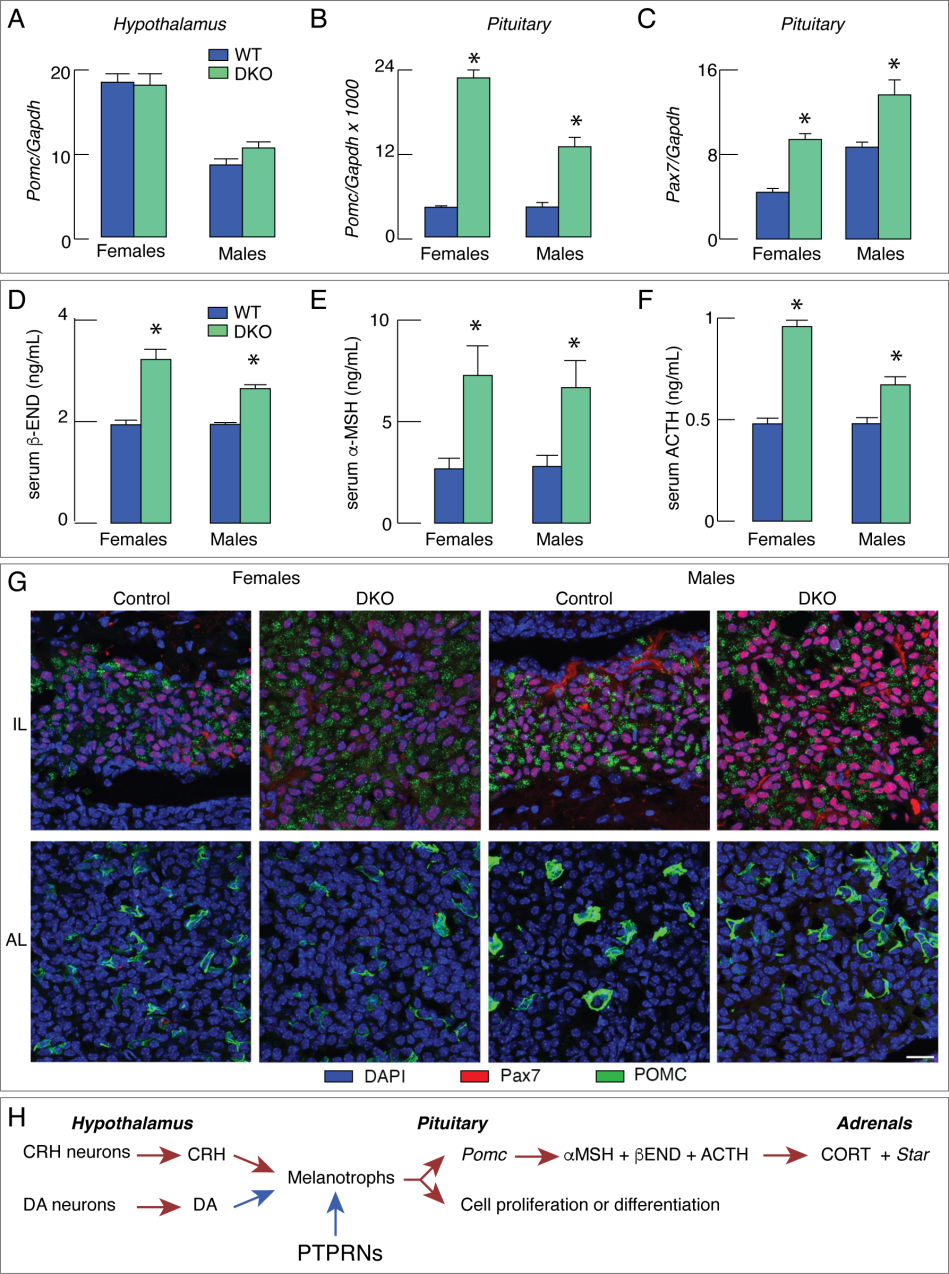
DKO increases pituitary melanotroph gene expression and hormone secretion, reflecting an increase in pituitary melanotroph population. **(A–F)** DKO does not affect *Pomc* expression in hypothalamus **(A)** but stimulates expression of this gene in pituitary **(B)** as well as *Pax7* expression in pituitary **(C)**. **(D–F)** Increase in *Pomc* expression was accompanied by elevation in serum hormone concentration: beta-endorphin (β-END; **D**), alpha-melanocyte stimulating hormone (α−MSH; **E**) and adrenocorticotropic hormone (ACTH; **F**). **(G)** Representative images of PAX7 (a maker protein of melanotrophs) and POMC immunostaining in intermediate lobe (IL) and anterior lobe (AL) of control and DKO female and male mice. The PAX7-immunopositive cells (red) were detected in IL, while POMC immunoreactivity (green) was present in both AL and IL of control and DKO animals. Note that IL of DKO mice of both sexes was larger than those from controls. The scale bar of 20 µm applies to all panels. **(H)** Schematic representation of the proposed inhibitory effect of PTPRNs on the hypothalamic-pituitary-gonadal axis by reducing *Pomc* expression and slowing melanotroph proliferation/differentiation. Red arrows - stimulation; blue arrows – inhibition. Asterisks indicate significant differences between pairs, P < 0.01.

A marker gene for the hypothalamic-pituitary-adrenal axis is *Pomc*, which is expressed in both the hypothalamus and the pituitary gland. In the hypothalamus, *Pomc* is expressed in the arcuate nucleus (66) and in the pituitary *Pomc* is expressed in corticotrophs and melanotrophs (67). DKO did not affect *Pomc* expression in the hypothalamus (**Figure 2A**), but dramatically increased expression in the pituitary (**Figure 2B**), although both tissues express PTPRNs. *Pomc* regulatory sequences in the pituitary and hypothalamic tissues differ (68), indicating that the tissue-specific PTPRNs actions are transcriptionally related. Single knockouts of *Ptprn* and *Ptprn2* also increased *Pomc* expression, but of smaller amplitudes compared to DKO (69).

Pituitary expression of *Pomc* is stimulated by hypothalamic corticotropin-releasing hormone (68), which is a ligand for corticotropin-releasing hormone receptor 1 expressed in corticotrophs and melanotrophs (67). However, DKO did not affect *Crh* expression (69). *Tbx19* is a common developmental transcription factor gene for corticotrophs and melanotrophs, whereas *Pax7* is expressed only in melanotroph (67) and ts expression in the pituitary was significantly elevated in DKO females and males (**Figure 2C**). TBX19 controls terminal differentiation of both lineages and, in cooperation with PITX1, activates *Pomc* transcription (70). PAX7 controls melanotroph differentiation (71) and facilitates TBX19-controlled *Pomc* transcription via chromatin remodeling (72).

DKO-increased *Pomc* expression in the pituitary gland was associated with increased hormone secretion *in vivo* and *in vitro*. Serum beta-endorphin was significantly elevated in both DKO females and males (**Figure 2D**), as were serum concentrations of alpha-melanocyte stimulating hormone (**Figure 2E**) and adrenocorticotropic hormone (**Figure 2F**). In cultured pituitary cells, both hormone release and cellular content of these hormones were significantly elevated, further indicating that *Ptprn* and *Ptprn2* regulate their synthesis ad release. DKO also increased serum corticosterone concentration, adrenal mass, and gene expression of the steroidogenic enzyme *Star* in adrenal tissue in both sexes (69).

Elevated expression of *Pax7* in the DKO pituitary is consistent with the hypothesis that *Pomc* expression and hormone synthesis and release are elevated in melanotrophs. The hypothesis was additionally confirmed by the finding that the expression of melanotroph-specific genes *Pcsk2*, *Esm1*, *Doc2g*, and *Oacyl* was also increased in DKO pituitaries. In contrast, there was no increase in the expression of the corticotrophs-specific genes *Chrna1*, *Clrn1*, *Trdn*, and *Hspb3* (69). Finally, immunohistochemical analysis using a POMC/adrenocorticotropic hormone-specific antibody to identify corticotrophs and melanotrophs and PAX7-specific antibody to label melanotrophs, showed that the intermediate lobe (home to melanotrophs) was enlarged, reflecting an increase in the population size of DKO melanotrophs. In contrast, we failed to detect an increase in the number of corticotrophs in the anterior lobe (**Figure 2G**). Therefore, both melanotrophs. hyperplasia and increased *Pomc* expression per cell in DKO mice are responsible for the significant increase in POMC-derived hormones. The scheme shown in **Figure 2H** illustrates the interpretation of these findings.

## Conclusions

A review of the PTPRN literature suggests an important conclusion; all neuroendocrine cells express PTPRN genes, but their knockout disrupts the function of only some of these cell types, suggesting a cell type-specific role for these pseudophosphatases. In the diffuse neuroendocrine system, the cells themselves control their own secretion, gene expression, and proliferation in response to stimulation. Thus, the role of PTPRNs in their function is more readily elucidated. In pancreatic β-cells, PTPRNs have been suggested to be an integral part of a system for monitoring β-cell-stimulated secretory activity and for adjusting insulin expression and initiating cell proliferation. There has also been significant progress in characterizing the molecular mechanism of action of PTPRNs in the exocytotic pathway, insulin gene expression, and cell proliferation. A growing number of reports also indicate that PTPRNs are involved in the tumorigenesis, but their mechanism of action has not been proven. The role of PTPRNs in other diffuse neuroendocrine cells has not been studied.

In the centralized neuroendocrine system organized as the hypothalamic-pituitary-target organ axes, there is a complex relationship as the participating cells synchronize their activity through feedforward and feedback mechanisms. It is therefore more difficult to identify the cell type directly affected by DKO, as demonstrated in work with the hypothalamic-pituitary-gonadal axis in female and male mice. Although PTPRNs are expressed in kisspeptinergic and GnRH neurons, as well as in gonadotrophs, only kisspeptinergic neurons in both regions of the hypothalamus have been identified as PTPRNs-responsive cell types, suggesting that stimulation of *Kiss1* expression increases hormone gene expression and secretion downstream of the axis (**Figure 1E**). In contrast, in the hypothalamic-pituitary-adrenal axis, melanotrophs have been identified as directly responding cells. Moreover, PTPRNs appear to inhibit both *Pomc* expression and melanotroph proliferation/differentiation, in parallel with the action of dopamine (**Figure 2G**). Further studies are needed to characterize the role of PTPRNs in the function of other hypothalamic and pituitary cells.

It is known that transcription factors can act as activators and repressors in different cells, but it is currently unknown whether PTPRNs act as transcription factors or upstream elements in the control of gene transcription. Further studies are needed to elucidate the molecular mechanism of this process. The cellular specificity of the PTPRNs actions is consistent with the specificity of promoter activation and repression for different genes, as suggested by the failure of DKO to increase *Pomc* expression in the hypothalamus but facilitate *Pomc* expression in the pituitary. The role of PTPRNs in normal and carcinoma cell proliferation also requires further studies.
